# *Leishmania* mortality in sand fly blood meal is not species-specific and does not result from direct effect of proteinases

**DOI:** 10.1186/s13071-018-2613-2

**Published:** 2018-01-15

**Authors:** Katerina Pruzinova, Jovana Sadlova, Jitka Myskova, Tereza Lestinova, Jozef Janda, Petr Volf

**Affiliations:** 1 Department of Parasitology, Faculty of Science, Charles University, Prague, Czech Republic; 2 Cytometry, Faculty of Science, Charles University, Prague, Czech Republic

**Keywords:** Sand fly, *Phlebotomus*, *Sergentomyia*, *Leishmania donovani*, *Leishmania major*, Blood meal digestion, Proteases

## Abstract

**Background:**

*Leishmania* development in sand flies is confined to the alimentary tract and is closely connected with blood meal digestion. Previously, it has been published that activities of sand fly midgut proteases are harmful to *Leishmania*, especially to amastigote-promastigote transition forms. However, our experiments with various *Leishmania*-sand fly pairs gave quite opposite results.

**Methods:**

We evaluated the effect of semi-digested midgut content on different life stages of *Leishmania donovani* and *Leishmania major* *in vitro*. Various morphological forms of parasites, including macrophage-derived amastigotes and transition forms, were incubated 2 h with midguts dissected at various intervals (6–72 h) post-blood meal or with commercially available proteinase, and their viability was determined using flow cytometry. In parallel, using amastigote-initiated experimental infections, we compared development of *L. donovani* in sand flies that are either susceptible (*Phlebotomus argentipes* and *P. orientalis*) or refractory (*P. papatasi* and *Sergentomyia schwetzi*) to this parasite.

**Results:**

*In vitro*, sand fly midgut homogenates affected *L. major* and *L. donovani* in a similar way; in all sand fly species, the most significant mortality effect was observed by the end of the blood meal digestion process. Surprisingly, the most susceptible *Leishmania* stages were promastigotes, while mortality of transforming parasites and amastigotes was significantly lower. Parasites were also susceptible to killing by rabbit blood in combination with proteinase, but resistant to proteinase itself. In vivo, *L. donovani* developed late-stage infections in both natural vectors; in *P. argentipes* the development was much faster than in *P. orientalis*. On the other hand, in refractory species *P. papatasi* and *S*. *schwetzi*, promastigotes survived activity of digestive enzymes but were lost during defecation.

**Conclusions:**

We demonstrated that *Leishmania* transition forms are more resistant to the killing effect of semi-digested blood meal than 24 h-old promastigotes. Data suggest that *Leishmania* mortality is not caused directly by sand fly proteases, we assume that this mortality results from toxic products of blood meal digestion. Survival of *L. donovani* promastigotes in refractory sand flies until blood meal defecation, together with similar mortality of *Leishmania* parasites incubated *in vitro* with midgut homogenates of susceptible as well as refractory species, contradict the previously raised hypotheses about the role of midgut proteases in sand fly vector competence to *Leishmania*.

**Electronic supplementary material:**

The online version of this article (10.1186/s13071-018-2613-2) contains supplementary material, which is available to authorized users.

## Background

Protozoan parasites of the genus *Leishmania* (Kinetoplastida: Trypanosomatidae) have a digenetic life-cycle consisting of extracellular promastigotes developing in sand fly vectors (Diptera: Phlebotominae) and intracellular amastigotes in mammalian hosts [1, 2]. *Leishmania donovani* causes human visceral leishmaniasis (VL, kala-azar), which is a deadly disease occurring mainly in the Indian subcontinent and East Africa. The most important vectors of this parasite are *Phlebotomus argentipes* and *P. orientalis* [3, 4]. *Leishmania major*, which is mainly transmitted by *P. papatasi* and *P. duboscqi*, is the causative agent of cutaneous leishmaniasis (CL), and is widely distributed in arid and savannah areas of sub-Saharan and North Africa, Middle East and Indian subcontinent [4, 5].

Various sand fly species differ in vector competence to various *Leishmania* species, and the knowledge of factors affecting vector competence is crucial from an epidemiological point of view [2, 6]. *Leishmania* development in sand flies is confined to the alimentary tract and is closely connected with blood meal digestion [1, 2]. As the majority of nutrients in the ingested blood is constituted by protein molecules, the proteolytic enzymes play the main role during blood meal digestion of sand flies, most of them being serine proteases, namely trypsin- and chymotrypsin-like molecules [7–9]. The midgut epithelial cells start to produce trypsin- and chymotrypsin-like proteases after the engorgement of the blood meal, activity levels are significantly increased from 6 h post-blood meal (pbm) and peak at 12–48 h pbm, depending on sand fly species [10–13].

It has been published repeatedly that proteolytic activities in the sand fly midgut affect *Leishmania* development [14–16]. According to Schlein & Romano [17] and Borovsky & Schlein [14], the specific components of trypsin-like activity cause the reduction of parasite numbers, or even death, of *L. donovani* promastigotes in the “non-compatible” vector *P. papatasi*, whereas the ability to influence this factor allows *L. major* to survive and develop within its natural vector. Using experimental infections of sand flies by promastigotes and the exogenous suppression of midgut proteolytic activity by soybean trypsin inhibitor, same authors [14, 16] established the hypothesis that activities of midgut proteases influence the vector competence of sand flies. Moreover, Pimenta et al. [15] have shown that the susceptibility of *Leishmania* parasites to destruction by midgut proteolytic activity in the natural vector is stage-specific. In their *in vitro* experiments, amastigotes and fully-transformed promastigotes of *L. major* were relatively resistant to *P. papatasi* proteases, while the parasites within amastigote-promastigote transition were highly susceptible to be killed.

In the present study, we evaluated the killing effects of midgut homogenates of various sand fly species on different morphological forms of *L. donovani* and *L. major*. Then, we use the most susceptible form, promastigotes, to study dynamics of this killing activity in four different sand fly species differing in susceptibility to *L. donovani*: two natural vectors, *P. argentipes* and *P. orientalis* [4, 12, 18], and two refractory ones, *P. papatasi* and *Sergentomyia schwetzi* [14, 16, 19, 20]. In parallel experiments *in vivo*, we compared *L. donovani* development in four sand fly species mentioned above, focussing on timing of parasite loss in refractory species.

## Methods

### Sand flies

Colonies of *P. argentipes*, *P. orientalis*, *P. papatasi* and *S. schwetzi* were maintained under standard conditions as described previously [21]. Three to seven day old females were fed on anesthetized mice, maintained at 26 °C and their midguts were dissected 6, 24, 32, 48 and 72 h pbm.

### *Leishmania* parasites and macrophages

*Leishmania donovani* promastigotes transfected with green fluorescence protein-GFP (MHOM/ET/2010/DM-1033/GR374) or red fluorescence protein-RFP (MHOM/ET/2009/AM459) and *L. major* promastigotes expressing dsRed fluorescence protein (MHOM/IR/−/173) were maintained at 23 °C in Medium 199 (Sigma-Aldrich, St. Louis, USA), supplemented with 10% foetal calf serum (Thermo Fisher Scientific, Waltham, USA), 1% BME vitamins (Sigma-Aldrich), 2% human urine and 250 μg/ml amikacin (Amikin, Medopharm, Prague, Czech Republic). *Leishmania donovani* promastigotes transfected with green and red fluorescence protein were maintained under pressure of selective antibiotic G 418 (150 μg/ml; Sigma-Aldrich) and hygromycin B (150 μg/ml; Sigma-Aldrich), respectively.

Amastigote stages of *L. donovani* were grown in J744 macrophage cell lines originating from BALB/c mice. Amastigotes of *L. major* were grown in bone-marrow macrophages (BMMs) differentiated from precursor cells of BALB/c mice in the presence of L929 fibroblast cell culture supernatant as a source of macrophage colony stimulating factor (M-CSF). Macrophages were exposed to stationary-phase promastigotes at a parasite to macrophage ratio of 8 promastigotes to 1 macrophage in 24-well plates (1 ml/well). Both infected and uninfected macrophages were cultured in complete RPMI-1640 medium (Sigma-Aldrich), containing 10% FBS, 1% penicillin-streptomycin (Sigma-Aldrich), 2 mM of L-glutamine (Sigma-Aldrich) and 0.05 mM of β-mercapto-ethanol at 37 °C with 5% CO_2._

We used macrophage-derived amastigotes in order to keep the 3R rules (refinement-replacement-reduction) and decrease the number of animals used in experiments. In comparison with tissue amastigotes, those isolated from the *in vitro* culture system were shown to be similarly infective to laboratory animals [22].

### Promastigote transformation assay

After 72–96 h post-infection, infected macrophages were disrupted by 0.016% SDS (Sigma-Aldrich) for 5–10 min and lysed cells were scraped by plunger from 1 ml insulin syringe. *Leishmania donovani* or *major* amastigotes were released to the Medium 199 and were allowed to transform at 23 °C. Three different *Leishmania* stages were used for *in vitro* experiments: amastigotes (immediately released from macrophages), parasites within amastigote-promastigote transition (5 h after the release from macrophages) and promastigote-like form (24 h after the release from macrophages). Determination of *Leishmania* stages was established on the basis of observation under the microscope; on 0 h all parasites were amastigotes, on 5 h there were transient forms with very small flagella and on 24 h 100 % of parasites in culture were promastigotes. This timing of parasite transformation was similar to Pimenta et al. [15], who used tissue amastigotes.

### Incubation of *Leishmania* with sand fly midgut homogenates and blood derivatives

Sand fly females were fed on anesthetized mice and dissected at different times post-blood meal (pbm). Dissection of midguts was semi-sterile and performed on ice, the preparation of one sample took maximum 30 min and the samples were immediately placed in a freezer (-80 °C). In experiments, midguts were homogenised while defrosting, centrifuged (12,000× *g*) and the supernatant was incubated with parasites.

In the first series of experiments, various *L. donovani* (GFP) or *L. major* (dsRed) stages were incubated with *P. argentipes*, *P. orientalis*, *P. papatasi* or *S. schwetzi* midgut homogenates (dissected 24 h pbm) for 2 h in a microwell plate (1000 *Leishmania*/midgut). In the second series of the experiments, *L. donovani* (GFP) promastigotes (24 h after the release from macrophages) were incubated with *P. argentipes*, *P. orientalis*, *P. papatasi* or *S. schwetzi* midgut homogenates (dissected 6, 24, 32, 48 and 72 h pbm) for 2 h in a microwell plate (1000 *Leishmania*/midgut). In addition, promastigotes 24 h post-transition were incubated with proteinase K (6.7 mg/ml, 2 U/mg protein; Roche, Mannheim, Germany), rabbit blood, red cells of rabbit blood, human haemoglobin (220 mg/ml; Sigma-Aldrich), rabbit blood + proteinase K, red cells + proteinase K and haemoglobin + proteinase K. *Leishmania* parasites incubated for 2 h with saline instead of midgut homogenates were used as a negative control and parasites killed by 1% formaldehyde and permeabilised by 0.5% Triton X-100 (Sigma-Aldrich) were used as a positive control.

After the incubation, parasites with midgut homogenate were transferred to saline solution, dead cell were marked with DAPI (4′,6-Diamidine-2′-phenylindole dihydrochloride, 0.005 mg/ml; Thermo Fisher Scientific) and analysed by flow cytometry. Flow cytometry measurements were performed using flow cytometer CytoFLEX S (Beckman Coulter, Inc., Brea, California, USA) equipped with 4 lasers (405 nm, 488 nm, 561 nm and 638 nm) and 13 fluorescence detectors. GFP was excited using 488 nm laser and its fluorescence emission was detected using 525/40 filter, dsRed was excited using 561 nm laser and its fluorescence emission was detected using 585/42 bandpass filter, DAPI was excited by 405 nm laser and detected using 450/50 filter. Analysis of cytometry data was performed using CytExpert software (Beckman Coulter).

The experiments were conducted in duplets and repeated twice. Statistical evaluations were performed by the ANOVA and *post-hoc* Tukey HSD test using SPSS Statistics 23.0 software.

### Sand fly infections

For amastigote-initiated infections, *L. donovani* (RFP) were co-cultivated with mouse macrophage line J774 for 72 h and non-internalized parasites were removed by thorough washing with pre-heated culture medium. Numbers of amastigotes per macrophages were established by fluorescent microscopy. The macrophages were removed from the culture plates by extensive washing with cold saline, centrifuged at 300× *g*, 4 °C for 10 min and resuspended in heat-inactivated rabbit blood for sand fly infections at the concentration of 1 × 10^6^ amastigotes/ml.

Females were dissected at several time intervals post-blood meal (pbm) and the abundance and location of *Leishmania* infections in the sand fly digestive tract were examined by fluorescent microscopy. Parasite loads were graded according to Myskova et al. [23] as light (< 100 parasites per gut), moderate (100–1000 parasites per gut) and heavy (> 1000 parasites per gut). Experiments with each *Leishmania*-sand fly combination were repeated four to six times.

### Morphometry of parasites

Midgut smears of sand flies infected with *Leishmania* parasites were fixed with methanol, stained with Giemsa, examined under an Olympus BX51 light microscope and photographed with an Olympus D70 camera. Body length, flagellar length and body width of parasites were measured using Image-J software. Four morphological forms were distinguished, as described in Sadlova et al. [24]: procyclic promastigotes (PP), elongated nectomonads (EN), metacyclic promastigotes (MP) and short promastigotes (SP). Haptomonads were not distinguished as representation of these attached forms is in principle underestimated on gut smears. In total, 160 randomly selected promastigotes from four females/smears for each sand fly species and time pbm were measured for comparison of amastigote-initiated *L. donovani* infections in four sand fly species.

## Results

### Effects of sand fly midgut homogenates on various forms of *L. donovani* and *L. major*

**Fig. 1:**
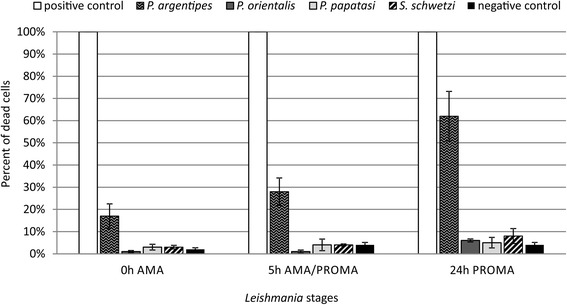
Different *L. donovani* stages incubated with sand fly midguts dissected at 24 h post-blood meal. *Leishmania donovani* amastigotes (0 h AMA), parasites within amastigote-promastigote transition (5 h AMA/PROMA) and promastigotes (24 h PROMA) were incubated in a microtiter plate for 2 h with saline or midguts of *P. argentipes*, *P. orientalis*, *P. papatasi* and *S. schwetzi* dissected at 24 h post-blood meal. As a positive control, parasites killed by 1% formaldehyde and permeabilised by 0.5% Triton X-100 were used

**Fig. 2:**
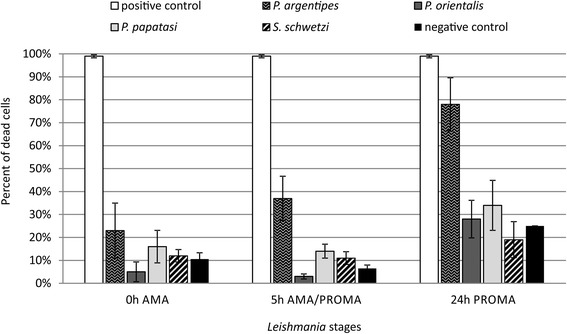
Different *L. major* stages incubated with sand fly midguts dissected at 24 h post-blood meal. *Leishmania major* amastigotes (0 h AMA), parasites within amastigote-promastigote transition (5 h AMA/PROMA) and promastigotes (24 h PROMA) were incubated in a microtiter plate for 2 h with saline or midguts of *P. argentipes*, *P. orientalis*, *P. papatasi* and *S. schwetzi* dissected at 24 h post-blood meal. As a positive control, parasites killed by 1% formaldehyde and permeabilised by 0.5% Triton X-100 were used

First we tested the killing effect of midgut homogenates from four sand fly species dissected 24 h pbm. All parasite forms tested (amastigotes, transition forms and promastigotes) of both *Leishmania* species were relatively resistant to killing effect of midgut homogenates of *P. orientalis*, *P. papatasi* and *S. schwetzi*; the mortality ranged between 1 and 8% in *L. donovani* (Fig. 1), 5–30% in *L. major* (Fig. 2) and was comparable to negative controls. Conversely, significant killing effect was caused by midgut homogenates of *P. argentipes*; the most susceptible forms were promastigotes with mortality about 60% (*L. donovani*) and 78% (*L. major*), mortality of transition forms and amastigotes was significantly lower, 28–37% and 17–22%, respectively (Tukey HSD, *P* < 0.001; Figs. 1 and 2). Representative images of flow cytometry analysis are shown in Additional file 1: Figures S1-S7.

### Effect of midgut homogenates from different sand fly species on *L. donovani* promastigotes

**Fig. 3:**
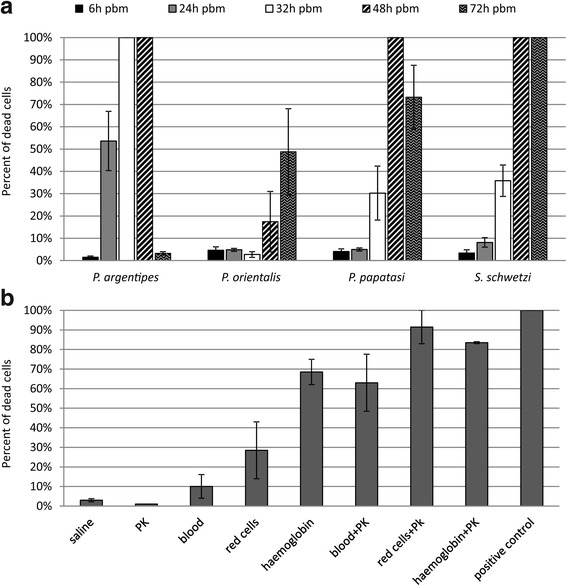
Promastigotes of *L. donovani* incubated with sand fly midguts dissected at different times post-blood meal and various controls. *Leishmania donovani* promastigotes (24 h after the releasing from macrophages) were incubated in a microtiter plate for 2 h with: **a** midguts of *P. argentipes*, *P. orientalis*, *P. papatasi* and *S. schwetzi* dissected at 6, 24, 32, 48 and 72 h post-blood meal; or **b** with saline, proteinase K (PK), rabbit blood, red cells of rabbit blood, human haemoglobin, blood + proteinase K, red cells + proteinase K and human haemoglobin + proteinase K. As a positive control, parasites killed by 1% formaldehyde and permeabilised by 0.5% Triton X-100 were used

The most susceptible stages, promastigotes (24 h after the release from the macrophages) were tested with sand fly midgut homogenates dissected at different times pbm (6, 24, 32, 48 and 72 h). Results are shown in Fig. 3a. Significant differences found in killing effect of various homogenates and time intervals (ANOVA, *F*_(6,27)_ = 834, *P* < 0.0001) correlated with differences in the time course of blood meal digestion: *Phlebotomus argentipes* digest fast and, therefore, significant promastigote mortality (Tukey HSD, *P* < 0.0001) was observed in groups incubated with homogenates obtained at 24 h pbm (mortality 55%), or 32 and 48 h pbm (mortality 100%). In contrast, *P. orientalis* digest slowly and significantly increased killing effect (Tukey HSD, *P* < 0.0001) was observed for homogenates dissected 72 h pbm (mortality 50%,). For *P. papatasi* and *S. schwetzi*, significantly enhanced mortality (Tukey HSD: *P* < 0.0001) was observed in promastigotes incubated with midguts dissected 32, 48 and 72 h pbm. As a negative control, we used midguts dissected 6 h pbm which did not cause any mortality of parasites in any of sand fly species (Fig. 3a).

In addition, we tested the effect of proteinase K and blood or blood derivatives on promastigotes. The results are shown in Fig. 3b. Proteinase K itself had no effect on parasites (mortality 1%), blood partly killed *Leishmania* (mortality 10%) while blood with addition of proteinase K caused significantly higher mortality (60%,Tukey HSD, *P* < 0.0001). A similar enhancing effect was observed in red cells, which killed about 30% of parasites, while red cells in combination with proteinase K caused significantly higher mortality of parasites (90%, Tukey HSD, *P* < 0.0001). Haemoglobin itself killed about 70% of *Leishmania* and haemoglobin with proteinase K caused mortality about 85%; this difference was not significant (Tukey HSD, *P* = 0.903).

### Comparison of *L. donovani* development in four vector species

The development of *L. donovani* was compared in detail in two natural vectors (*P. argentipes* and *P. orientalis*) and two refractory species (*P. papatasi* and *S. schwetzi*), using amastigote-initiated experimental infections of sand flies.

**Fig. 4:**
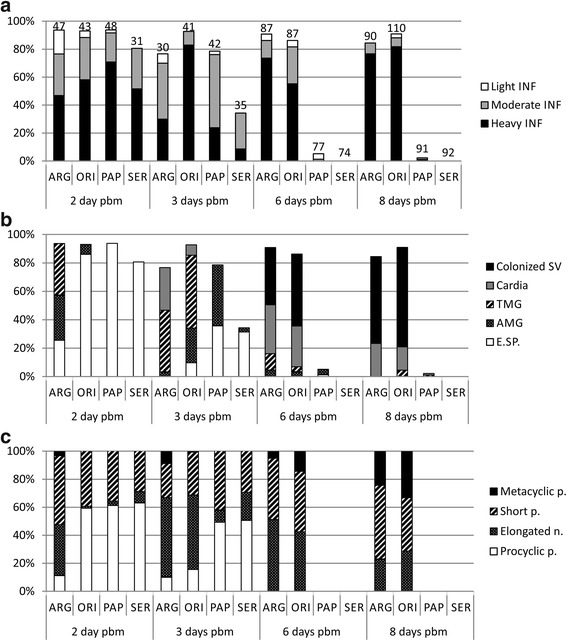
Amastigote-initiated infections of *L. donovani* in four sand fly species: *P. orientalis* (ORI), *P. argentipes* (ARG), *P. papatasi* (PAP) and *S. schwetzi* (SER). **a** Rates and intensities of *L. donovani* infections. Numbers of dissected females are shown above bars. Differences between species were evaluated using Chi-square test: day 2 PBM, *χ*^2^ = 20.009, *df* = 9, *P* = 0.018; day 3 pbm, *χ*^2^ = 74.880, *df* = 9, *P* < 0.0001; day 6 pbm, *χ*^2^ = 255.6, *df* = 9, *P* < 0.0001; day 10 pbm, *χ*^2^ = 296.2, *df* = 9, *P* < 0.0001. **b** Location of *L. donovani* in infected sand flies. Differences between species were evaluated using Chi-square test: day 2 PBM, *χ*^2^ = 96.863, *df* = 9, *P* < 0.0001; day 3 pbm, *χ*^2^ = 121.7, *df* = 12, *P* < 0.0001; day 6 pbm, *χ*^2^ = 260.1, *df* = 15, *P* < 0.0001; day 10 pbm, *χ*^2^ = 301.6, *df* = 9, *P* < 0.0001. **c** Morphological forms of *L. donovani* in infected sand flies. The guts of infected females were sampled at 2, 3, 6 and 10 days pbm and parasite morphometry determined as described in methods. The percentage of each form found in infected flies at each time point is shown. Differences among lines were most significant during early infections; day 2 pbm, *χ*^2^ = 190.7, *df* = 9, *P* < 0.0001; day 3 pbm, *χ*^2^ = 196.5, *df* = 9, *P* < 0.0001; day 6 pbm, *χ*^2^ = 8.572, *df* = 2, *P =* 0.014; day 10 pbm, *χ*^2^ = 6.755, *df* = 2, *P* = 0. 034. *Abbreviations*: E.SP., endoperitrophic space, AMG, abdominal midgut; TMG, thoracic midgut; SV, stomodeal valve

Before defecation, by day 2 pbm, the infection rates were similar in all four sand fly species: more than 80% of females were infected and heavy or moderate intensities of infections prevailed (Fig. 4a). However, the location of parasites differed among sand fly species. In all *P. papatasi* and *S. schwetzi*, as well as in 93% of *P. orientalis*, infections were still enclosed inside the peritrophic matrix (PM). Conversely, in *P. argentipes*, most infections reached the abdominal midgut (AMG) or even the thoracic midgut (TMG) (Fig. 4b). Faster development of *L. donovani* in *P. argentipes* was reflected by higher proportion of elongated nectomonads in comparison with prevailing procyclic forms in other three sand fly species (Fig. 4c).

By day 3 pbm, the pattern of *Leishmania* development highly differed among sand fly species studied. In *P. orientalis*, both rates and intensities of infections were very high and parasites were found in a wide range of locations, either still remaining with digested blood in the endoperitrophic space (11%) or reaching the AMG (26%) or the TMG (55%) or even the cardia region (8%). In *P. argentipes*, 83% of females already defecated blood remnants and most parasites reached the TMG (57%) or even the cardia region (39%). Importantly, in *P. papatasi*, no parasites were found in females which already defecated (19%). In non-defecated females, living parasites were either enclosed inside the endoperitrophic space (45%) or reached the AMG (55%). Similarly, in *S. schwetzi*, defecated females were parasite-free (66%), in non-defecated individuals, parasites were enclosed inside the PM. While elongated nectomonads prevailed in proven vectors and metacyclic forms were also already present, procyclic promastigotes remained the prevailing form in both refractory species (Fig. 4c).

Late-phase infections differed considerably among the four sand fly species. In *P. orientalis* and *P. argentipes* (proven vectors of *L. donovani*), parasites developed heavy infections, infection rates did not fall below 80% and colonization of the stomodeal valve (SV) was observed in 59% and 44% of infected females by day 6 pbm, and 77% and 72% by day 10 PBM, respectively (Fig. 4a, b). Proportion of metacyclic promastigotes did not differ significantly between *P. orientalis* and *P. argentipes* (Fig. 4c). On the other hand, infection rates in *P. papatasi* rapidly decreased to 5% and 2% by day 6 and 10 pbm, respectively, and in those few positive females promastigotes never colonized the SV. No *Leishmania* infection was detected in *S. schwetzi* after defecation.

## Discussion

More than two decades ago it was proposed that (i) activities of sand fly midgut proteases are harmful to *Leishmania* in non-compatible vectors [14, 16, 17], and (ii) in natural vectors, the most susceptible stages to digestive enzymes are forms in transition from amastigotes to promastigotes [15]. In the present study, we tested both these hypotheses and evaluated the effect of sand fly midgut digestion on various *L. donovani* and *L. major* stages in vivo as well as in vitro.

Interestingly, for both parasite species tested, *L. donovani* and *L. major*, promastigotes were found to be the most susceptible forms to killing by midgut homogenates *in vitro*, while mortalities of transition forms and amastigotes were significantly lower. These findings are in contrast with the study of Pimenta et al. [15], where authors reported 95% reduction in numbers of viable transforming parasites, but fresh amastigotes and 18 h-old promastigotes were less susceptible to be killed.

Incubation of *L. donovani* promastigotes with midgut homogenates dissected from various sand fly species at different times pbm supported the fact that effect of blood digestion on parasites is not selective and species-specific. The mortality of parasites was more influenced by the time pbm of used midgut homogenates than by the sand fly species. The four sand fly species substantially differ in kinetics of blood meal digestion [12], which was well reflected in time-course of the harmful effect of midgut homogenates on *L. donovani* in these experiments. In susceptible as well as refractory parasite-vector combinations, the mortality of parasites increased with the degree of digested blood and the highest mortality effect was observed at the end of their blood meal digestion.

Experiments *in vitro* also showed susceptibility of *Leishmania* parasites to human haemoglobin or combination of rabbit blood or red blood cells with proteinase, but demonstrated their resistance to proteinase itself. As haemoglobin negatively influences the development of infective *Leishmania* promastigotes in culture [25], and haem generated after haemoglobin hydrolysis is well known for its cytotoxicity [26], we assume that *Leishmania* are destructed by products of blood meal digestion, like haem and/or reactive oxygen radical generated by free Fe^2+^, but not directly by midgut proteases as was postulated before [14–16]. *Leishmania* parasites can profit from inhibition or delay of midgut proteolytic activities by soybean trypsin inhibitor, as was shown in previous studies [14, 16, 27], because it prolongs the parasites hospitable environment without toxic products of blood meal digestion. It was also observed that sand fly midguts without blood meal have no negative effect on *Leishmania* in medium. In the study of Doehl et al. [28], promastigotes with added midgut homogenates dissected 12 days pbm grew similarly to negative controls.

Previous studies showed that *P. argentipes* and *P. orientalis* are similarly susceptible to experimental infection with Ethiopian strain of *L. donovani* and even one or two *Leishmania* promastigotes are sufficient for the establishment of mature late-stage infection [12, 18]. In this study, both natural vectors of *L. donovani* also provided excellent conditions for the development of *L. donovani* parasites after infection by amastigotes, but the kinetics of infections substantially differed. The complete development of *L. donovani* in *P. argentipes* was extremely fast; parasites escaped from the endoperitrophic space by day 1 pbm, reached the cardia region by day 2 pbm and colonized the stomodeal valve by day 4 pbm. In contrast, in *P. orientalis*, *Leishmania* reached all these aforementioned midgut regions with two-day delay. This striking difference is caused by a very fast blood meal digestion in *P. argentipes* females in comparison with *P. orientalis*. It was recently shown that the peak of proteolytic activities, PM degradation and defecation of blood meal remnants came one to 3 days earlier in *P. argentipes* than *P. orientalis* [12].

As expected, in refractory species *P. papatasi* and *S. schwetzi*, *L. donovani* did not establish late stage infections. However, importantly, *L. donovani* amastigotes transformed to promastigotes which developed and multiplied in sand fly midguts until defecation.

High or even 100% mortality of promastigotes exposed to digested midguts of *P. argentipes* and *P. orientalis* midguts *in vitro* contradicts successful *L. donovani* development in both natural vectors. One must consider specific conditions accompanying blood digestion in hematophagous insects, where haem generated from haemoglobin catabolism is bound to the peritrophic matrix, which provides its detoxification and protects *Leishmania* against haem toxicity [29]. In addition, in natural conditions promastigotes escape into the ectoperitrophic space and colonise the abdominal midgut of sand flies at time corresponding to periods when the killing effect on *Leishmania* *in vitro* was highest (48 and 72 h pbm); therefore, they escaped from direct contact with toxic radicals released at the end of blood meal digestion.

Refractoriness of *P. papatasi* to *L. donovani* has been attributed to two different mechanisms. The first hypothesis has suggested that intestinal trypsin-like activity itself prevents development of *L. donovani* in *P. papatasi* [14, 16]. The second hypothesis stressed that the vectoral competence in *P. papatasi* correlates with the ability of promastigotes to attach to the sand fly gut [30] and LPG-mediated binding of promastigotes to galectin on midgut epithelium was proposed as the key factor restricting susceptibility of *P. papatasi* to *L. major* [30, 31] and *L. turanica* [32, 33]. Our results support the second hypothesis as *L. donovani* parasites survived the peak of activity of digestive enzymes, and were able to escape from the PM and colonize the abdominal midgut. Loss of parasites came up later and coincided with the passage of the blood remnants.

## Conclusions

We showed that transition forms of *Leishmania* are less susceptible to killing effect of semi-digested blood meal (24 h pbm) than promastigotes and we assume that sand fly midgut proteases do not play important roles in refractoriness of *P. papatasi* to *L. donovani*. The mortality of parasites observed *in vitro* was unspecific, occurs in natural parasite-vector pairs as well in refractory combinations and increased with the degree of digested blood. *In vivo*, viable *L. donovani* promastigotes survived and multiplied in *P. papatasi* until the moment of defecation, which confirms insufficiency of *L. donovani* to bind to *P. papatasi* midgut [30]. Midgut binding, not midgut proteases, are therefore responsible for refractoriness of *P. papatasi* to *L. donovani*.

## Additional file

Additional file 1: Figure S1. Gating strategy. a Gating on single GFP+ cells on GFP-A/GFP-H dotplot. b Gating on *Leishmania* cells on FSC-A/SSC-A dotplot. c Analysis of DAPI fluorescence on histogram – enumeration of % DAPI+ (i.e. dead) *Leishmania* cells. **Figure S2.** Different *Leishmania donovani* stages  were incubated with midguts of *P. argentipes* dissected at 24 h post-blood meal. Analysis of dead *Leishmania* was performed using flow cytometry. Percentage of dead cells was assessed on histogram of DAPI fluorescence on gated single GFP-positive *Leishmania*. **Figure S3.** Different *Leishmania donovani* stages were incubated with midguts of *P. orientalis* dissected at 24 h post-blood meal. Analysis of dead *Leishmania* was performed using flow cytometry. Percentage of dead cells was assessed on histogram of DAPI fluorescence on gated single GFP-positive *Leishmania*. **Figure S4.** Different *Leishmania donovani* stages were incubated with midguts of *P. papatasi* dissected at 24 h post-blood meal. Analysis of dead *Leishmania* was performed using flow cytometry. Percentage of dead cells was assessed on histogram of DAPI fluorescence on gated single GFP-positive *Leishmania*. **Figure S5.** Different *Leishmania donovani* stages were incubated with midguts of *S. schwetzi* dissected at 24 h post-blood meal. Analysis of dead *Leishmania* was performed using flow cytometry. Percentage of dead cells was assessed on histogram of DAPI fluorescence on gated single GFP-positive *Leishmania*. **Figure S6.** Negative control; different *Leishmania donovani* stages were incubated with saline. Analysis of dead *Leishmania* was performed using flow cytometry. Percentage of dead cells was assessed on histogram of DAPI fluorescence on gated single GFP-positive *Leishmania*. **Figure S7.** Positive control; parasites killed by 1% formaldehyde and permeabilised by 0.5% Triton X-100. Percentage of dead cells was assessed on histogram of DAPI fluorescence on gated single GFP-positive *Leishmania*. (PPTX 346 kb)

## Abbreviations

AMG: Abdominal midgut
CL: Cutaneous leishmaniasis
DAPI: 4′,6-Diamidine-2′-phenylindole dihydrochloride
EN: Elongated nectomonads
GFP: Green fluorescence protein
M-CSF: Macrophage colony stimulating factor
MP: Metacyclic promastigotes
pbm: Post-blood meal
PM: Peritrophic matrix
PP: Procyclic promastigotes
RFP: Red fluorescence protein
SP: Short promastigotes
SV: Stomodeal valve
TMG: Thoracic midgut
VL: Visceral leishmaniasis
