# Supplementary material for: Leishmania mortality in sand fly blood meal is not species-specific and does not result from direct effect of proteinases
Source: Parasit Vectors. 2018 Jan 15;11:37. doi: 10.1186/s13071-018-2613-2 (PMC5769529; doi:10.1186/s13071-018-2613-2)
Supplement: Additional file 1: Figure S1. — Gating strategy. a Gating on single GFP+ cells on GFP-A/GFP-H dotplot. b Gating on Leishmania cells on FSC-A/SSC-A dotplot. c Analysis of DAPI fluorescence on histogram – enumeration of % DAPI+ (i.e. dead) Leishmania cells. Figure S2. Different Leishmania donovani stages were incubated with midguts of P. argentipes dissected at 24 h post-blood meal. Analysis of dead Leishmania was performed using flow cytometry. Percentage of dead cells was assessed on histogram of DAPI fluorescence on gated single GFP-positive Leishmania. Figure S3. Different Leishmania donovani stages were incubated with midguts of P. orientalis dissected at 24 h post-blood meal. Analysis of dead Leishmania was performed using flow cytometry. Percentage of dead cells was assessed on histogram of DAPI fluorescence on gated single GFP-positive Leishmania. Figure S4. Different Leishmania donovani stages were incubated with midguts of P. papatasi dissected at 24 h post-blood meal. Analysis of dead Leishmania was performed using flow cytometry. Percentage of dead cells was assessed on histogram of DAPI fluorescence on gated single GFP-positive Leishmania. Figure S5. Different Leishmania donovani stages were incubated with midguts of S. schwetzi dissected at 24 h post-blood meal. Analysis of dead Leishmania was performed using flow cytometry. Percentage of dead cells was assessed on histogram of DAPI fluorescence on gated single GFP-positive Leishmania. Figure S6. Negative control; different Leishmania donovani stages were incubated with saline. Analysis of dead Leishmania was performed using flow cytometry. Percentage of dead cells was assessed on histogram of DAPI fluorescence on gated single GFP-positive Leishmania. Figure S7. Positive control; parasites killed by 1% formaldehyde and permeabilised by 0.5% Triton X-100. Percentage of dead cells was assessed on histogram of DAPI fluorescence on gated single GFP-positive Leishmania. (PPTX 346 kb) [file 13071_2018_2613_MOESM1_ESM.pptx]

## Slide 1
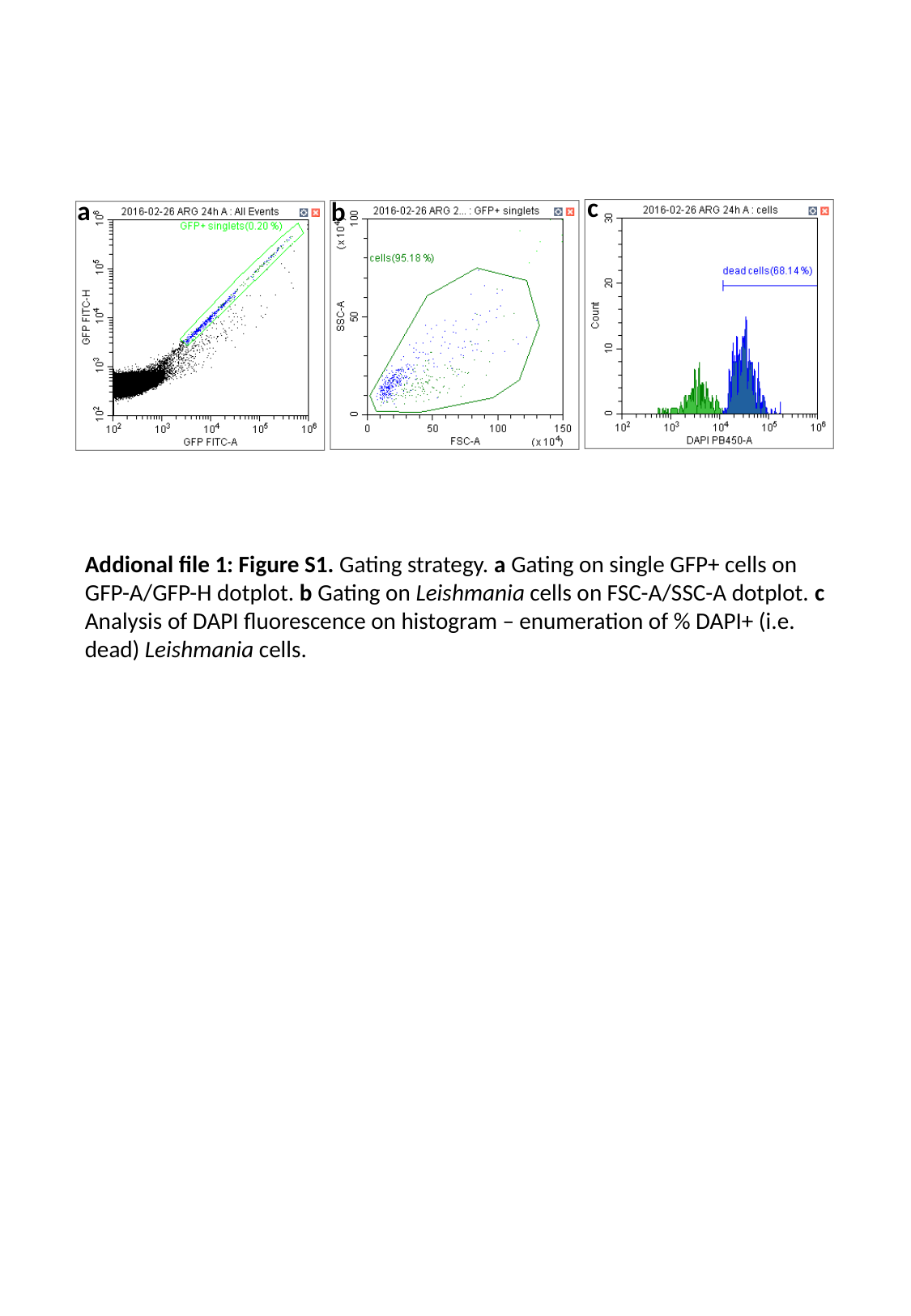

c
a
b
Addional file 1: Figure S1. Gating strategy. a Gating on single GFP+ cells on GFP-A/GFP-H dotplot. b Gating on Leishmania cells on FSC-A/SSC-A dotplot. c Analysis of DAPI fluorescence on histogram – enumeration of % DAPI+ (i.e. dead) Leishmania cells.

## Slide 2
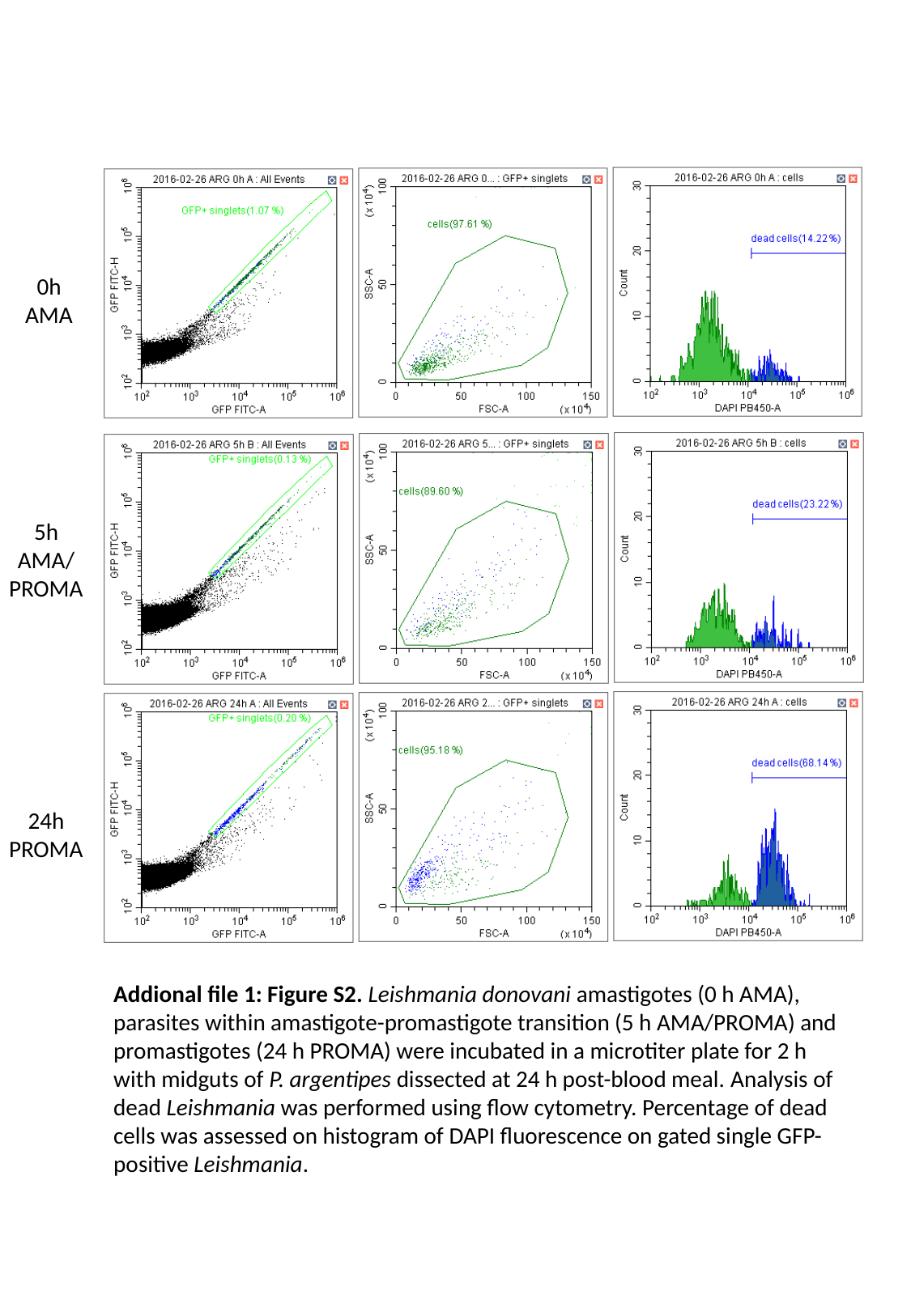

0h
AMA
5h
AMA/
PROMA
24h
PROMA
Addional file 1: Figure S2. Leishmania donovani amastigotes (0 h AMA), parasites within amastigote-promastigote transition (5 h AMA/PROMA) and promastigotes (24 h PROMA) were incubated in a microtiter plate for 2 h with midguts of P. argentipes dissected at 24 h post-blood meal. Analysis of dead Leishmania was performed using flow cytometry. Percentage of dead cells was assessed on histogram of DAPI fluorescence on gated single GFP-positive Leishmania.

## Slide 3
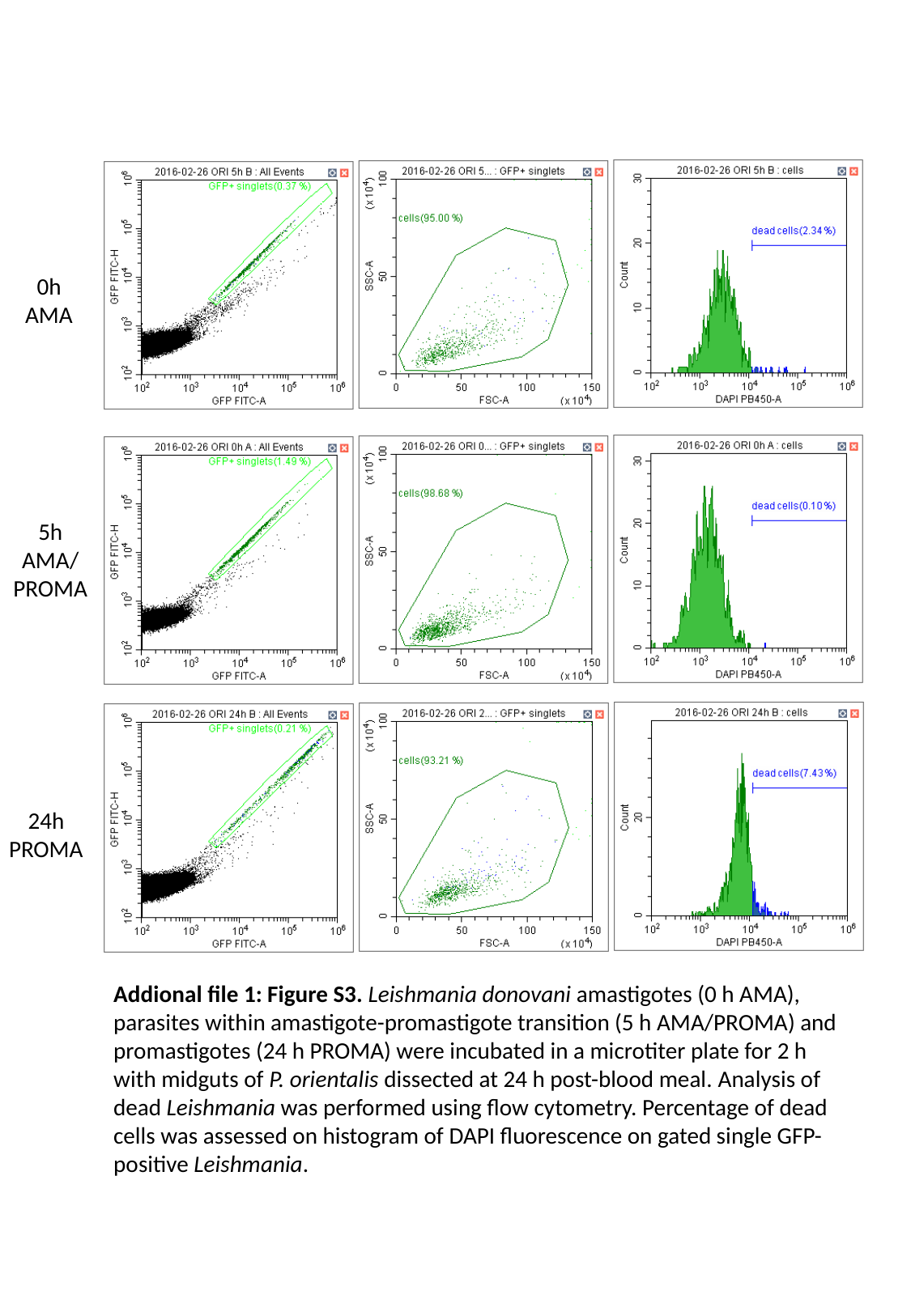

0h
AMA
5h
AMA/
PROMA
24h
PROMA
Addional file 1: Figure S3. Leishmania donovani amastigotes (0 h AMA), parasites within amastigote-promastigote transition (5 h AMA/PROMA) and promastigotes (24 h PROMA) were incubated in a microtiter plate for 2 h with midguts of P. orientalis dissected at 24 h post-blood meal. Analysis of dead Leishmania was performed using flow cytometry. Percentage of dead cells was assessed on histogram of DAPI fluorescence on gated single GFP-positive Leishmania.

## Slide 4
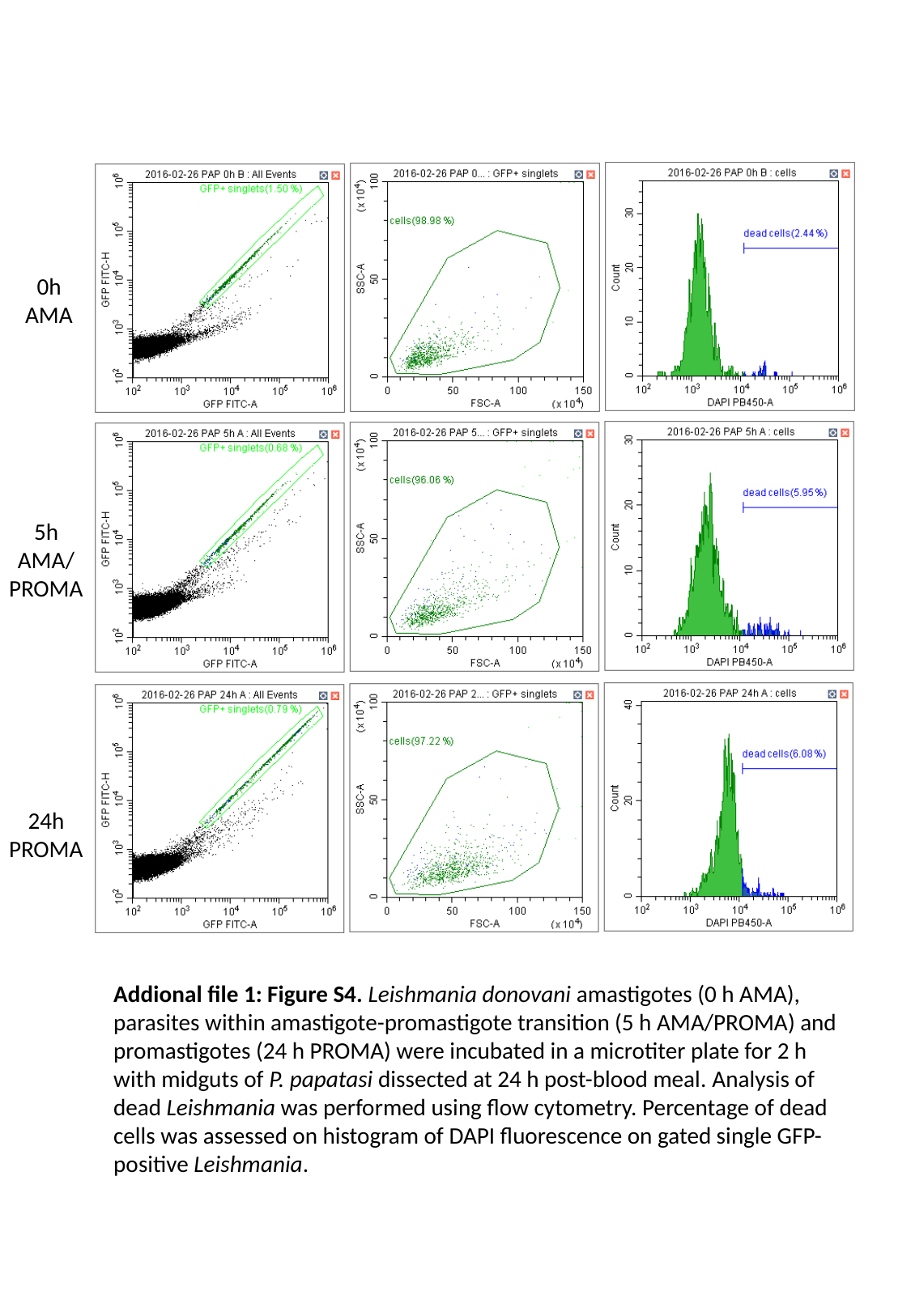

0h
AMA
5h
AMA/
PROMA
24h
PROMA
Addional file 1: Figure S4. Leishmania donovani amastigotes (0 h AMA), parasites within amastigote-promastigote transition (5 h AMA/PROMA) and promastigotes (24 h PROMA) were incubated in a microtiter plate for 2 h with midguts of P. papatasi dissected at 24 h post-blood meal. Analysis of dead Leishmania was performed using flow cytometry. Percentage of dead cells was assessed on histogram of DAPI fluorescence on gated single GFP-positive Leishmania.

## Slide 5
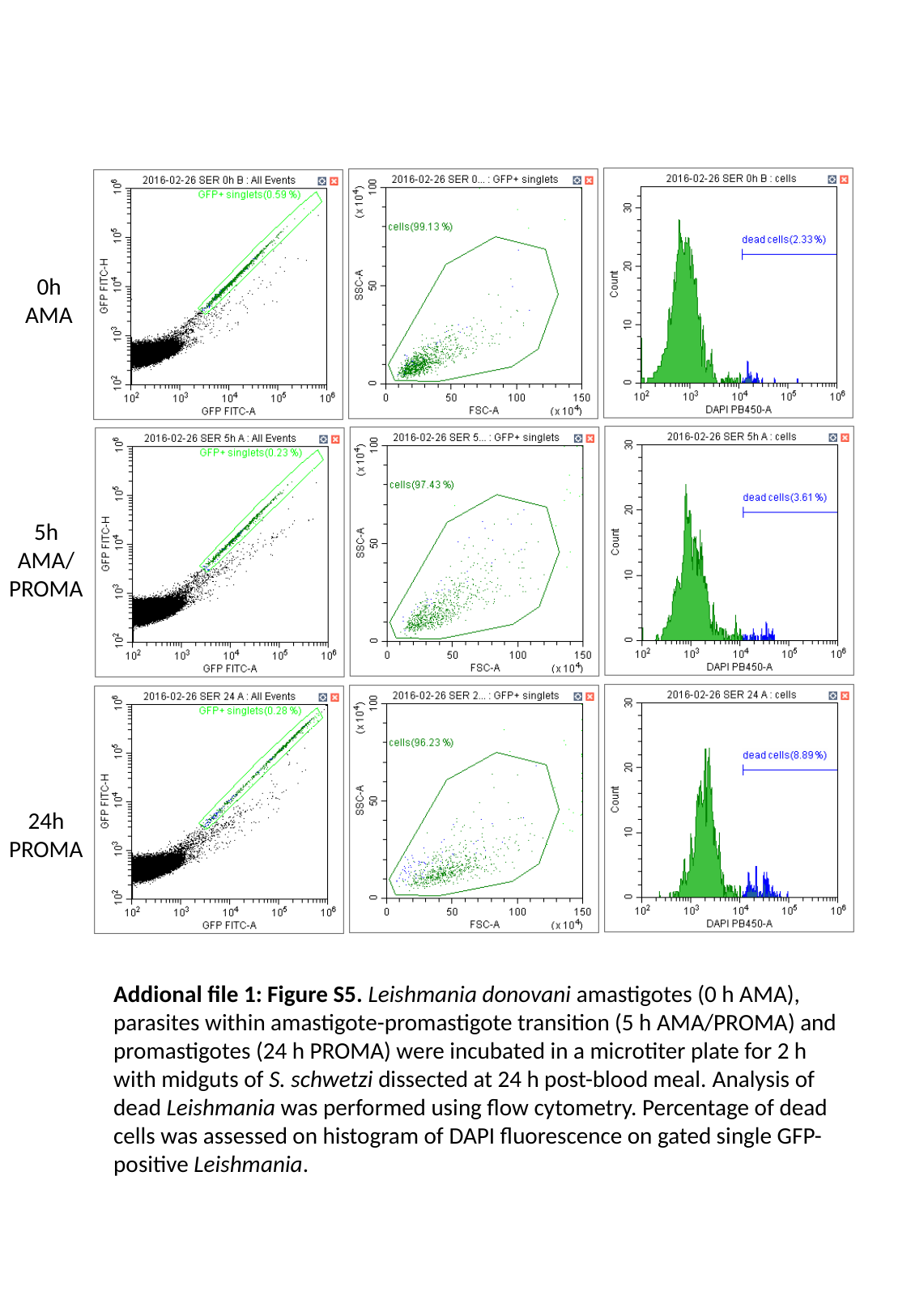

0h
AMA
5h
AMA/
PROMA
24h
PROMA
Addional file 1: Figure S5. Leishmania donovani amastigotes (0 h AMA), parasites within amastigote-promastigote transition (5 h AMA/PROMA) and promastigotes (24 h PROMA) were incubated in a microtiter plate for 2 h with midguts of S. schwetzi dissected at 24 h post-blood meal. Analysis of dead Leishmania was performed using flow cytometry. Percentage of dead cells was assessed on histogram of DAPI fluorescence on gated single GFP-positive Leishmania.

## Slide 6
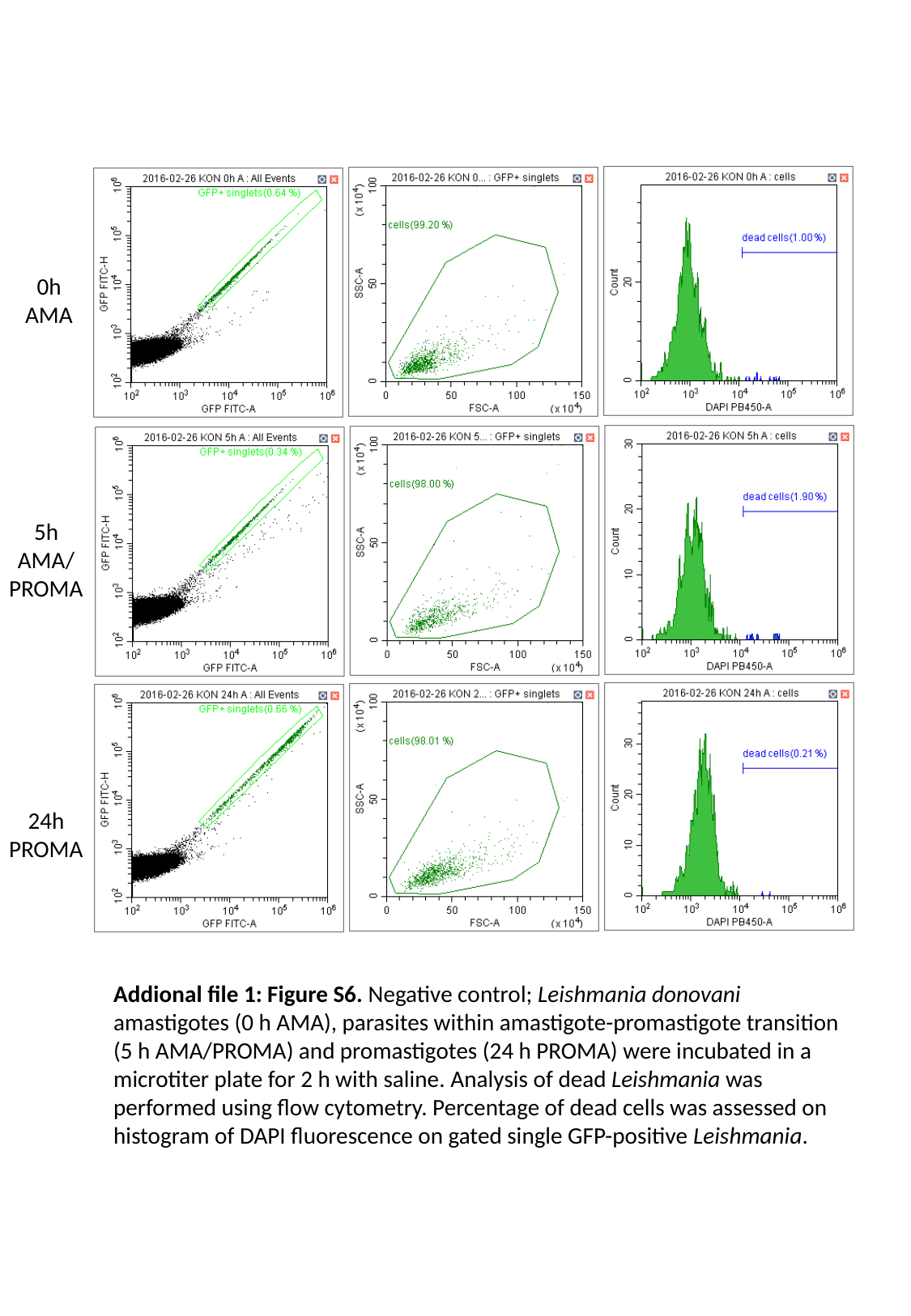

0h
AMA
5h
AMA/
PROMA
24h
PROMA
Addional file 1: Figure S6. Negative control; Leishmania donovani amastigotes (0 h AMA), parasites within amastigote-promastigote transition (5 h AMA/PROMA) and promastigotes (24 h PROMA) were incubated in a microtiter plate for 2 h with saline. Analysis of dead Leishmania was performed using flow cytometry. Percentage of dead cells was assessed on histogram of DAPI fluorescence on gated single GFP-positive Leishmania.

## Slide 7
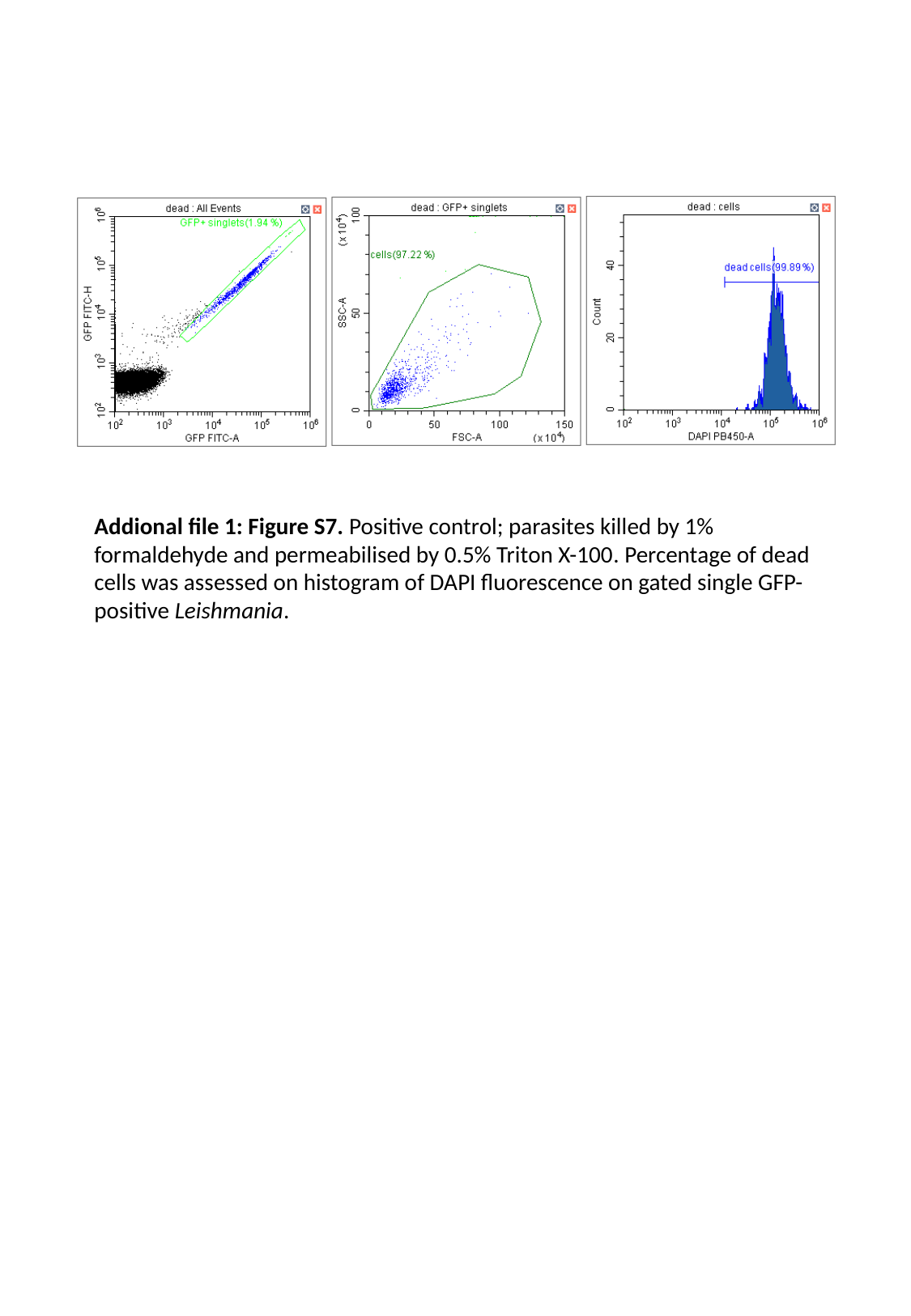

Addional file 1: Figure S7. Positive control; parasites killed by 1% formaldehyde and permeabilised by 0.5% Triton X-100. Percentage of dead cells was assessed on histogram of DAPI fluorescence on gated single GFP-positive Leishmania.
